# New instrumentation in percutaneous nephrolithotomy

**DOI:** 10.4103/0970-1591.70579

**Published:** 2010

**Authors:** Joseph W. Pugh, Benjamin K. Canales

**Affiliations:** Department of Urology, University of Florida, Gainesville, FL, USA

**Keywords:** Percutaneous nephrolithotomy, lithotripsy, stone manipulation devices, tract sealants

## Abstract

Percutaneous nephrolithotomy (PCNL) is the procedure of choice for removing large, complex, and/or multiple renal calculi. Since its first description in 1976, PCNL techniques and equipment have evolved to maximize procedural efficacy, safety, and reproducibility. We reviewed current literature from January 2004 to November 2009 using Medline search regarding PCNL instrumentation and technology. Additional equipment discovered during the review process without published Medline evidence was summarized from manufacturer brochures and data. Included in this review are summaries of intracorporeal lithotriptors and accessory equipment, stone manipulation devices, PCNL tract sealants, and a digital rigid nephroscope. The evolution of these devices from their predecessors has increased the instrumentation options for the treating urologist and may represent more effective technology for the percutaneous treatment of large renal stones.

## INTRODUCTION

Percutaneous nephrolithotomy (PCNL), first described in 1976,[1] has become the procedure of choice for the removal of kidney stones larger than 2 cm[2] or 1.5 cm located within the lower pole.[2–4] Equipment associated with this technique has evolved to increase stone free rates, to decrease operative time, and to reduce patient morbidity and mortality. We performed a literature search using Medline from January 2004 to November 2009 regarding published studies on PCNL equipment and products. In this review, we focus on the literature for lithotripsy devices (Gyrus ACMI CyberWand^®^, Swiss LithoClast Select with Vario^®^ and LithoPUMP^®^, Cook LMA StoneBreaker^®^), digital nephroscopes (Olympus Invisio^®^ Smith), stone manipulation devices (PercSys Accordion^®^, Cook Perc N-Circle^®^), and sealants/matrix products following tubeless PCNL.

## INTRACORPOREAL LITHOTRIPTORS

Ultrasonic and pneumatic lithotriptors are the most commonly used energy source for percutaneous lithotripsy, fragmenting stones with high success rates and minimal soft tissue effects.[5–7] Ultrasonic lithotriptors, first described for fragmentation of bladder stones,[8] transmit acoustic wave energy along a probe that is converted at the probe tip to mechanical energy. Because the energy is based on vibration and not heat or shockwaves, efficient stone fragmentation requires direct stone contact with this instrument type. Small refinements have been made in ultrasonic lithotripsy technology over the last 10-15 years, but in 2007, a “dual” ultrasonic lithotriptor was introduced that holds tremendous promise.

### 

#### CyberWand^®^

**Figure 1:**
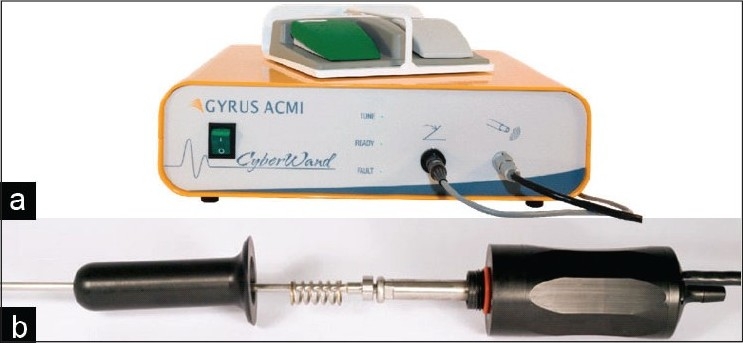
CyberWand^®^ dual probe ultrasonic lithotripter with dual energy foot pedal sitting atop generator (a.) and disassembled handpiece (b.). Note: parts on handpiece from left to right include spring, floating probe, free ring, and fixed probe

The Gyrus ACMI CyberWand^®^ [Figure 1] is considered a dual ultrasonic lithotriptor because it employs two separate ultrasonic probes that vibrate at two different (high and low) frequencies via one hand-piece. The synergistic effect of both probes vibrating at different rates is thought to account for its efficacy. The inner probe, designed for larger stones, vibrates at 21,000 Hz and measures 2.77 mm (outer diameter) with a 2.1 mm hollow inner lumen. This lumen is connected directly through the hand-piece to a suction tube which exits in-line with the probe (an advantage compared to other hand-piece types that come off at a variety of angles). The outer probe is 3.75 mm in diameter, vibrates at 1000 Hz, and is designed for breaking smaller stones. It is approximately one mm shorter than the inner probe and is thought to have some ballistic effect on stones. Both probes are controlled with one foot pedal, giving the surgeon the choice of energy modality. To date, three *in vitro* and one clinical (non-outcome) studies have been published for this device.

Kim *et al*.[9] compared fragmentation rates of the CyberWand^®^ and LithoClast Master^®^ on gypsum stones in a hands-free, *in vitro* testing system. The stone was sandwiched between a 63.4 gm weight and lithotriptor probe tip inside a translucent acrylic sheath, providing constant force between the stone and probe. Both devices were calibrated to the maximal manufacturer settings, and ten stones were treated. The CyberWand^®^ had almost twice faster mean stone penetration time of 4.8 seconds compared to 8.1 seconds for the LithoClast Master^®^. Neither device overheated, became occluded, or malfunctioned during the experiment. Goldman and associates[10] used BegoStone phantoms hooked to a motorized water circulator pump to determine what factors (if any) affect efficiency between these devices. Varying probe contact pressures of 400, 1,000, and 2,000 gm were applied to stones, but regardless of LithoClast setting or probe combination, the CyberWand was able to extract more stone mass. No effect was noted by altering rotational range or frequency for either device. A third study[11] compared the new Vario^®^ handpiece with CyberWand^®^ and is discussed later in this section. Lastly, Soucy and colleagues[12] used a digital sound survey meter to measure decibel levels at the level of the urologist’s head during intracorporeal lithotripsy. The CyberWand^®^ ’s mean decibel level of 93 dB was significantly louder than an Olympus LUS-2^®^ ultrasonic lithtripter (65 dB) or a holmium laser fiber (60 dB). Overall, the CyberWand appears to be just as efficacious in the management of renal stones as its “single” ultrasonic predecessors. In laboratory testing, the dual probe offers more rapid stone penetration time in comparison to the LithoClast Ultra^®^. Clinical trials are needed to better quantify its benefits and define it uses in intracorporeal lithotripsy.

#### StoneBreaker^®^

Pneumatic lithotripsy technology utilizes a ballistic probe for stone fragmentation. The probe is connected to an air supply (compression tank) that creates pressure to propel it into a stone. The energy, similar to that of a jackhammer, causes stone fragmentation but has traditionally been limited due to the tedious manual extraction of single stone fragments despite suction devices. Not surprisingly, over the last 10 years, almost all reports of pneumatic lithotripsy have been reported in association with ultrasonic lithotriptors. The newest pneumatic device, the Cook LMA StoneBreaker^®^, is a portable pneumatic lithotriptor that is powered by a light-weight cartridge of high pressure carbon dioxide gas. One full cartridge allows for delivery of over 80 shocks while providing pressures up to 2.9 MPa at the probe tip. The device is portable, does not require an electrical outlet source, and has minimal tip movement during firing, allowing for safe stone fragmentation. Probe tips come in 1.0 F (50 cm, 60.5 cm), 1.6 F (50 cm, 60.5 cm), or 2 F (42.5 cm) with smaller probes being described for use during ureteroscopy.

Only one clinical study has been reported to date for this device. Rane and Kommu performed a multi-center prospective study of the StoneBreaker^®^ in 102 stone patients who required intracorporeal lithotripsy.[13] Of those patients, 49 were PCNL, 48 were ureteroscopy (using 1.0 F probe), and five were cystolithalopaxy. Specifically for PCNL, 33 cases were staghorn/partial staghorn, and 16 cases were renal pelvic stones. Mean (range) stone size was 2.8 (1.8-4.8) cm, and six patients (12%) required multiple punctures. Mean (range) number of shocks for complete fragmentation and subsequent removal was 34 (2-76), and all stone types were satisfactorily fragmented. KUB stone free rates were reported to be 100% with minimal evidence of urothelial trauma and or stone retropulsion. Overall, the StoneBreaker^®^ appears to be effective and safe for percutaneous lithotripsy use and has the added benefits of being light-weight, portable, and non-electric. Like most other devices in the pneumatic family, it is limited by the need for manual stone extraction or an associated suction device (like an ultrasonic lithotriptor). More trials are needed to fully determine the role and effectiveness of this device.

#### LithoClast Select^®^ with Vario^®^ and LithoPUMP^®^

**Figure 2:**
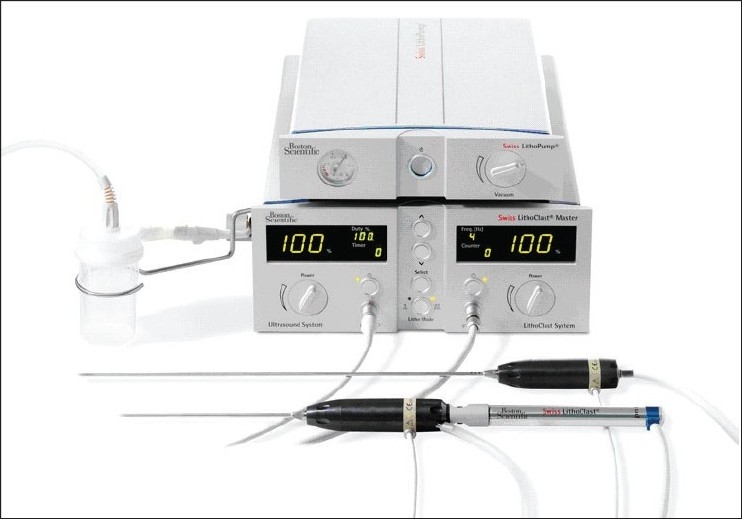
Swiss LithoPump ^®^ atop LithoClast Select^®^ base unit with two variations of handpieces. Vario^®^ hand piece (top) with straight suction connector for ultrasonic only mode and angled suction connection (bottom) for combination pneumatic and ultrasonic mode

The LithoClast Master^®^ (EMS, Nyon, Switzerland), known as the LithoClast Ultra^®^ (Boston Scientific, Natick, MA) in North America, is a popular combination ultrasonic and pneumatic lithotriptor available for many years. The most recent advance with this unit is the Vario^®^ ultrasonic handpiece and the LithoPUMP^®^ [Figure 2], marketed in the U.S. as the LithoClast Select^®^. The Vario^®^ handpiece has undergone tuning to optimize the output of its piezoceramic crystals at transducer frequencies of 23.2 to 26.4 kHz, which is believed to allow higher power output with longer probe life and less heating. Additionally, the handpiece has been redesigned with less of an acute angle when the pneumatic probe is in place and a fenestrated outlet at the tubing connection to minimize stone clogging. Only one trial (*in vitro*) has been reported for the Vario^®^ [Table 1]. Louie *et al*.[11] tested the CyberWand^®^ and LithoClast Master^®^ with Vario^®^ handpiece in a cystolithalopaxy model using BegoStones (hard) and Ultracal-30 stones (softer) in a simulated clinical scenario. The CyberWand^®^ fragmented the Ultracal-30 stones faster than the LithoClast Vario^®^ handpiece but failed on four separate occasions to break the harder BegoStones. Notably, the CyberWand probes repeatedly fractured at the probe solder joint, a design flaw that has been reportedly addressed in newer models. Clearance time of all fragments was similar for both products.

**Table 1:** Percutaneous lithotripsy and nephroscope products

Name	Description	Price USD*	Power Source*	PCNL Studies
Gyrus ACMI CyberWand^®^	Dual ultrasonic lithotriptor; 300 grams (0.66 lbs) plus probe	System: $36,000 Probe: $750	100-240V, 50/60Hz	Kim *et al*.[9]
				Goldman *et al*.[10]
				Louie *et al*.[11]
				Soucy *et al*.[12]
Swiss LithoClast Select^®^ with Vario^®^ and LithoPUMP^®^	Pneumatic and ultrasonic lithotripter; re-designed handpiece; adjustable footpedal suction device	New device: $45,000 Upgrade: $13,000	100-240V, 50/60Hz; Compressed air	Louie *et al*.[11]
Cook LMA StoneBreaker^®^	Pneumatic, 480 grams (1.06 lbs)	Handpiece: $10,000 Probe: $205 Cartridge: $15	CO2 cartridge	Rane and Kommu.[13]
Invisio^®^ Smith Digital Nephroscope	Digital rigid nephroscope; 470 grams (1.04 lbs)	Nephroscope: $15,000 Digital Controller: $17,500	Controller box 100-240V, 50/60 Hz	Andonian *et al*.[16]
				Borin *et al*.[17]
				Quayle *et al*.[18]

* Price and power source obtained from U.S. 2009 catalog listings and may vary by region or availability

Now, LithoClast Select^®^ and Master^®^ models also include the LithoPUMP^®^ device. When using standard ultrasonic lithotripsy, occasionally suction pressure will exceed irrigant flow, causing external air to be pulled into the sheath and collapse of the collecting system. This may impair vision during the procedure, may cause nuisance mucosal bleeding, or may cause the probe to overheat if prolonged. The LithoPUMP^®^ is an adjustable suction device that is activated only when the ultrasonic footpedal is pressed. This is thought to improve vision and reduce the potential for overheating the device. Overall, the original Swiss LithoClast product series has been well tested and time-proven, and the current additions seem to facilitate more efficient lithotripsy. A few head to head trials have been performed for these devices, but a multi-center clinical trial to evaluate and compare the speed and efficiency of stone fragmentation for each of these products is currently underway at the International Kidney Stone Institute, and preliminary results are expected soon.

## DIGITAL RIGID NEPHROSCOPES

Visualization of the renal pelvis and calyces are of paramount importance while performing PCNL. The Storz rigid nephroscope was one of the first tools used for this purpose,[14] and since its introduction in 1965, the fiber optic rod-lens nephroscope has been the standard for percutaneous renal surgery.[15] The Olympus Invisio^®^ Smith digital nephroscope integrates the endoscope, digital camera, and light source in one simple “plug and play” device,[16] similar to newly introduced digital ureteroscopes. The Invisio^®^ weighs almost half (470 gm, 1.04 lbs) that of a standard rod-lens nephroscope (939 gm, 2.07 lbs). The tip of the scope houses a 1 mm digital camera and dual LED driven light carriers, negating the need for an external xenon light source. Compared to fiberoptic scopes *in vitro*, the digital image has demonstrated higher resolution[17,18] and the 4.9 mm (15 F) working channel allows for insertion of a variety of instruments, forceps, lithotriptor probes (including the CyberWand^®^), or suction devices. Overall, this “next generation” of digital nephroscopes offers a lighter and more ergonomic design with higher image resolution compared to its fiber optic predecessors. Although promising, more trials are needed to justify the cost and benefit of this device and further define its use.

## STONE MANIPULATION DEVICES

Regardless of the energy source, there is always some degree or retropulsion during stone fragmentation. The Accordion^®^ Stone Management Device is a 2.9F, hydrophilic-coated, microcatheter-based tool which creates an occlusion to prevent retropulsion of stone fragments. Its efficacy at preventing retrograde fragment migration has been well described during ureteroscopic lithotripsy.[17,18] Wosnitzer *et al*. retrospectively evaluated the ability of the Accordion^®^ to prevent antegrade stone migration during PCNL[19] The authors retrospectively reviewed a PCNL database and compared PCNL surgery in 30 patients with the device (Accordion^®^ group) and 30 patients without the device (PCNL group) who were matched for stone size and composition. For the Accordion^®^ group, a ureteral catheter was placed thru a 17F cystoscope, and the device placed and deployed at the level of the ureteropelvic junction and proximal ureter. The Accordion^®^ was then tied to the ureteral catheter and foley to prevent migration. PCNL was then carried out in standard fashion mainly using ultrasonic lithotripsy. The Accordion^®^ device was successfully placed in all patients, and in all but one patient (3%), it prevented stone migration down the ureter. The Accordion^®^ group had 13 patients (43%) who required ureteral stenting at case end compared to 17 patients (57%) in the PCNL group. Although the findings had statistical significance, the clinical significance of four patients is difficult to interpret in a retrospective trial of this nature. However, for urologists who do not like to perform post-PCNL ureteroscopy, this device appears to be effective in preventing stone migration and could be considered similar to most standard ureteral balloon occlusion devices. It is not known if this device increases stone free rates or decreases OR times, and currently clinical trials are on-going in this area.

**Figure 3:**
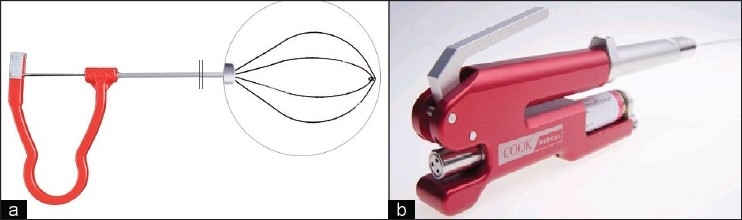
Cook Perc N-Circle^®^ (a.) with close-up inset of nitinol tipless basket and StoneBreaker^®^ (b.) handpiece with CO_2_ cartridge

The Cook Perc N-Circle^®^ is a handheld, tipless stone basket designed specifically for PCNL [Figure 3]. The 2 cm, tipless basket deploys off a 10 F, 39 cm lightweight probe by squeezing a handle. The basket design should be familiar to urologists who use N-Circle^®^ baskets during ureteroscopy as it allows for direct positioning against the mucosal lining with minimal trauma, reducing bleeding and maintaining a clear working field. The nitinol wire construction provides strength and flexibility for manipulation of kidney stones.

Hoffman *et al*. performed an *in vitro* study comparing the Storz 3-prong grasper and the Cook 12F Perc N-Circle^®^.[20] A three, five, and eight mm human calculus were placed in the calyx of a percutaneous renal model. A 26F Storz nephroscope was inserted through a 30F sheath. Operators were randomized to perform stone extraction with the two devices. Testing alternated between the two devices until ten extractions attempts were conducted. Time to extraction and number of inadvertent withdrawals of the sheath were recorded. Mean extraction time for the Perc N-Circle^®^ was 25.3 second and 35.1 for the 3-prong grasper. Inadvertent removal of the stone sheath occurred 7% of the attempts with the Perc N-Circle^®^ and 53% of the attempts with the 3-prong grasper. The Cook Perc N-Circle^®^ in this model was shown to be a more expedient at stone removal than the 3-prong grasper with less risk of sheath removal. As the amount of urothelial trauma could not be assessed in this study, clinical *in vivo* studies are needed to comment on the effectiveness of this device during PCNL.

## TUBELESS PCNL TRACT CLOSURE

Advances in PCNL equipment have also been associated with improved dilation, stone removal, and ending tube techniques. One of the more commonly employed techniques is the tubeless approach, where a ureteral stent instead of a nephrostomy tube is left in place at procedure end. Although this is a procedural advance, a variety of “sealants” and matrix-type products have been used in an attempt to improve tract hemostasis, decrease postoperative urinary tract leakage, and provide tissue support when a nephrostomy is not placed. Notably, there is a key distinction between matrix hemostats and the fibrin sealants. The matrix agents, such as FloSeal^™^, Surgicel^™^, Spongostan^™^ and Gelfoam^™^, require a bleeding source for fibrinogen, whereas the fibrin sealants, such as Tisseel^™^, do not. Thus, it has been suggested that matrix sealants would be best suited for obtaining adjunctive hemostasis rather than for tissue adhesion and sealing.[21,22] However, this should not preclude them from being studied in relation to tubeless PCNL.

### 

#### Sealants

Tisseel^™^ is a four-component product [Table 2] with both hemostatic and adhesive properties. It is mixed using a two-chamber device similar to epoxy glue and requires about 20 minutes of preparation time in order to set. Three clinical studies were identified for tubeless PCNL and Tisseel^™^ [Table 2]. Noller *et al*.[23] prospectively followed a group of 10 patients by postoperative CT and demonstrated no extravasation or urinomas.[23] Mikhail *et al*.[24] retrospectively compared two equally matched tubeless PCNL groups with and without Tisseel^™^. No differences were seen in either group for blood loss or pain control. Length of stay was shorter by about 17 hours in the experimental group with a *P*=0.05. Using a similar grouping, Shah and colleagues[25] performed a prospective randomized control trial [Table 2] with a Tisseal^™^ group of 32 and a control group of 31. There were no differences noted in hematocrit or blood transfusion, and the only significant variable was decreased postoperative pain and less analgesic use in the Tisseal^™^ group (*P*<0.05).

**Table 2:** Hemostatic agents used in PCNL and reported *in vivo* literature results

Product Name	Description; mechanism	Studies	Study design (n)	Significant variables (*P* value)
Spongostan^™^ (Johnson and Johnson)	Porcine-derived gelatin sponge; matrix for platelet aggregation, clot formation	Schick 2006[33]	Prospective series (7)	N/A
		Nagele 2007[34]	Prospective series (11)	N/A
		Singh 2008[26]	Prospective RCT (50)	Urine soaking, pain (<0.001)
		Singh 2009[35]	Prospective series (45)	N/A
Tisseel^™^ (Baxter)	Fibrinogen, thrombin, aprotinin, and calcium; tissue sealant, clot formation, hemostasis	Mikhail 2003[24]	Retrospective case control (40)	Length of stay (0.05)
		Noller 2004[23]	Prospective cohort (10)	No difference
		Shah 2006[25]	Prospective RCT (63)	Pain, analgesia use (<0.05)
Floseal^™^ (Baxter)	Bovine gelatin, human thrombin; matrix for platelet aggregation, clot formation	Lee 2004[29]	Retrospective case series (2)	N/A
		Schilling 2008[30]	Prospective case control (40)	No difference
Surgicel^™^ (Ethicon)	Oxidized cellulose; clot formation, hemostasis	Aghamir *et al*. 2006[32]	Prospective RCT (20)	No difference
Gelfoam^™^ (Pharmacia and Upjohn)	Porcine-derived gelatin sponge; matrix for platelet aggregation, clot formation	Yu 2006[27]	Prospective case control (30)	Less transfusion (0.013), pain (<0.01), length of stay (0.035)

N/A – Not applicable (study was not designed to compare groups)

#### Matrix agents

Spongostan^™^, a gelatin matrix sponge derived from porcine skin has been reported following tubeless PCNL in three different studies [Table 2]. Two case series document the safety of the product.[21,22] Singh *et al*.[26] performed a prospective randomized trial comparing two groups of similar patients following PCNL with or without Spongostan^™^. Significantly less pain, lower analgesia requirements, and less wound soakage (*P*<0.001) as well as a trend toward a shorter hospital stay (*P*<0.057) were observed in the gelatin-assisted tubeless group. A similar type of gelatin matrix, Gelfoam^™^, has similarly been tried in the post-PCNL setting with similar results.[27] No significant complications were noted during gelatin placement in any of these series [Table 2].

Floseal, a flowable gelatin hemostatic matrix, has also being used as a sealant following tubeless PCNL. Kaufmann and associates[28] describe a technique of balloon placement to decrease the chance of Floseal migration within the collecting system. Lee *et al*. first described the use of Floseal following tubeless PCNL in two patients following tubeless PCNL.[29] Both patients had stable postoperative hemoglobin and no evidence of bleeding or obstruction on postoperative computerized tomography.

Schilling and colleagues[30] performed a prospective case control study comparing similar groups of patient undergoing mini-PCNL. At case end, patients were randomized to “tubeless” with Floseal product or small bore nephrostomy tube. Total analgesic use and length of hospital stay was similar for both groups, and the only appreciable difference was a slightly higher stone free rate in Floseal group (95%) compared to nephrostomy (85%).

Surgicel has a wide variety of uses in urology that has been well described in the literature.[31] Aghamir *et al*.[32] published their randomized control trial results assessing the effectiveness of Surgicel as a sealant following tubeless PCNL [Table 2]. Twenty patients were randomized following tubeless PCNL into Surgicel (n=10) and controls. No statistically significant difference was seen for any study endpoints, including post-operative hematocrit changes, urinary extravasation by abdominal ultrasonography, or wound dressing inspection. Small sample may have played a role in this. Overall, no convincing arguments have been made by fairly well designed, prospective trials for the use of either matrix or sealants in the tubeless PCNL setting. Many of the trials discussed are limited by poor randomization methods and small numbers; so it remains to be seen if the trend in less post-operative pain remains true in further trials.

## CONCLUSION

A variety of new products and instruments have been introduced over the last five years for urologists who perform PCNL. The newer lithotriptors appear to be just as effective, if not more effective, than their predecessors at stone fragmentation. New stone retrieval and occlusion devices can be valuable tools in successful PCNL in the correct clinical setting. The Smith digital nephroscope provides clearly superior vision compared to its fiber-optic family members and should be considered for urologists with a large PCNL practice. However, no durability data exists for this device. Lastly, hemostatic and adhesive agents may hold great potential to seal tubeless PCNL tracts, but for now, the literature lacks power in this area.
